# A study of skin marker alignment using different diamond‐shaped light fields for prone breast external‐beam radiation therapy

**DOI:** 10.1002/acm2.13772

**Published:** 2022-08-27

**Authors:** Huijun Xu, Sally B. Cheston, Arun Gopal, Baoshe Zhang, Shifeng Chen, Suhong Yu, Andrea Hall, Sara Dudley

**Affiliations:** ^1^ Department of Radiation Oncology University of Maryland School of Medicine Baltimore Maryland USA; ^2^ Department of Radiation Oncology University of Massachusetts Memorial Medical Center Worcester Massachusetts USA

**Keywords:** breast cancer, diamond‐shaped light field, EBRT, patient setup alignment, prone position

## Abstract

For breast cancer patients treated in the prone position with tangential fields, a diamond‐shaped light field (DSLF) can be used to align with corresponding skin markers for image‐guided radiation therapy (IGRT). This study evaluates and compares the benefits of different DSLF setups.

Seventy‐one patients who underwent daily tangential kilovoltage (kV) IGRT were categorized retrospectively into four groups: (1) DSLF field size (FS) = 10 × 10 cm^2^, gantry angle = 90° (right breast)/270° (left breast), with the same isocenter as treatment tangential beams; (2) same as group 1, except DSLF FS = 4 × 4 cm^2^; (3) DSLF FS = 4 × 4–6 × 8 cm^2^, gantry angle = tangential treatment beam, off‐isocenter so that the DSLF was at the approximate breast center; and (4) No‐DSLF. We compared their total setup time (including any DSLF/marker‐based alignment and IGRT) and relative kV‐based couch shift corrections. For groups 1–3, DSLF‐only dose distributions (excluding kV‐based correction) were simulated by reversely shifting the couch positions from the computed tomography plans, which were assumed equivalent to the delivered dose when both DSLF and IGRT were used.

For patient groups 1–4, the average daily setup time was 2.6, 2.5, 5.0, and 8.3 min, respectively. Their mean and standard deviations of daily kV‐based couch shifts were 0.64 ± 0.4, 0.68 ± 0.3, 0.8 ± 0.6, and 1.0 ± 0.6 cm. The average target dose changes after excluding kV‐IGRT for groups 1–3 were−0.2%, −0.1%, and +0.4%, respectively, whereas DSLF‐1 was most efficient in sparing heart and chest wall, DSLF‐2 had lowest lung *D*_max_; and DSLF‐3 maintained the highest target coverage at the cost of highest OAR dose.

In general, the use of DSLF greatly reduces patient setup time and may result in smaller IGRT corrections. If IGRT is limited, different DSLF setups yield different target coverage and OAR dose sparing. Our findings will help DSLF setup optimization in the prone breast treatment setting.

## 1 INTRODUCTION

External‐beam radiation therapy (EBRT) is used in a majority of patients with breast cancer.^1^, ^2^ Because of the associated excellent local control rates, two opposing tangential beams are often used. In many clinics, prone positioning is preferred to the supine position to decrease the lung dose and the heart dose.^3^, ^4^, ^5^, ^6^, ^7^, ^8^, ^9^ In the prone position, gravity causes the breast to fall away from the chest wall, moving the target away from the lung and heart, as well as decreasing breast separation and improving dose homogeneity. Moreover, prone positioning results in less intrafractional variations^10^ due to the markedly reduced respiratory motion in the chest wall as well as clip motion.^11^, ^12^ Prone treatment, however, is associated with more uncertainties in patient position reproducibility. For example, when the isocenter is in the middle of the breast instead of the chest wall,^13^ there can be greater setup and interfractional variation.

To minimize the effects of the setup variations, imaging guidance is commonly adopted. In image‐guided radiation therapy (IGRT) for photon tangential treatment, megavoltage (MV) imaging is more widely used in general because of associated direct and faster anatomy alignment, whereas kilovoltage (kV) yields better contrast and lower imaging dose.^11^ Two‐dimensional planar imaging is usually performed, and three‐dimensional cone‐beam computed tomography (CBCT) may be an option.^12^ According to two recent studies,^13^, ^14^ daily kV‐ or MV‐based IGRT was recommended for left‐sided breast patients, whereas weekly kVs or MVs might be sufficient for right‐sided breast patients. However, frequent IGRT is not always beneficial. For example, concerns may include excessive imaging doses and patient tolerance for prolonged imaging/treatment time.

Therefore, daily skin marker alignment can play a key role in breast treatment setup, especially in the case of a limited X‐ray‐based IGRT scheme. The patient is positioned prone, with the breast situated approximately in the center of the opening of the breast board. Triangulation tattoos are commonly used based on a plane passing through the nipple and defined by laser lights at the time of simulation so that patient can be leveled against laser on a daily basis in the future treatment.^15^ In some clinics, a predefined diamond‐shaped light field (DSLF) projection has been introduced to further improve treatment alignment efficiency. Most of our clinical sites implement a 2‐step process for patient prone treatment setup, as Xu et al.^13^ described: First, a predefined DSLF is projected on the breast to match the corresponding patient skin marker, which is drawn based upon the corners and edges of the projection of the DSLF on the first day of treatment after kV/MV‐based IGRT setup. Then, a daily tangential kV‐based IGRT is performed, with a couch shift correction in the vertical and longitudinal directions to further match the breast contour, clips, and chest wall with the digitally reconstructed radiograph (DRR) at treatment beam's eye view.

Compared to the traditional body tattoo alignment, the DSLF setup is favored by our therapists because it helps them quickly reproduce the simulated treatment position based on matching the skin marker against a 2D light field projected on the breast in the prone position. Optical surface management systems such as Vision RT (Vision RT Ltd., London, United Kingdom), which can effectively reproduce supine breast patient setup,^16^, ^17^ are rarely used for prone breast treatment because of difficulties in surface matching. Therefore, it cannot replace the DSLF's role in the prone breast setup. Three DSLF techniques with different gantry angles and field sizes (FSs) have been developed and utilized across our treatment practices. Their benefits in comparison to No‐DSLF, with regard to the patient setup efficiency and particularly their dosimetric effects in scenarios of limited IGRT, have not been studied.

This work compares the practicality and dosimetric effects of these different DSLF setups based on their total patient setup time and kV‐based couch shifts. By analyzing differences in dose distribution between the DSLF‐ and IGRT‐based setups, we can calculate the relative dosimetric impacts of DSLF on target coverage and OAR sparing. This work aims to provide a reference for optimizing DSLF setups in the workflow of prone breast EBRT.

## 2 METHODS AND MATERIALS

Seventy‐one patients with early‐stage breast cancer treated in our institution were retrospectively studied in this work. Patients were prescribed 4256 cGy in 16 fractions to the right or left whole breast in the prone position using a pair of field‐in‐field tangential beams from linear accelerators such as Varian Trilogy and TrueBeam (Varian Medical Systems, Inc, Palo Alto, CA). Based on our institutional clinical practice guideline, the whole breast planning target volume (PTV) included the whole‐breast clinical target volume (CTV) plus a 0.5‐cm margin, with the exclusion of the rib and 0.5‐cm off skin. The CTV‐to‐PTV expansion for tumor bed was 1.0 cm for boost irradiation, excluding lung, heart, and 0.5‐cm off skin. Some prone breast treatments were followed by a 1000‐cGy boost supine treatment in four fractions.

**FIGURE 1:**
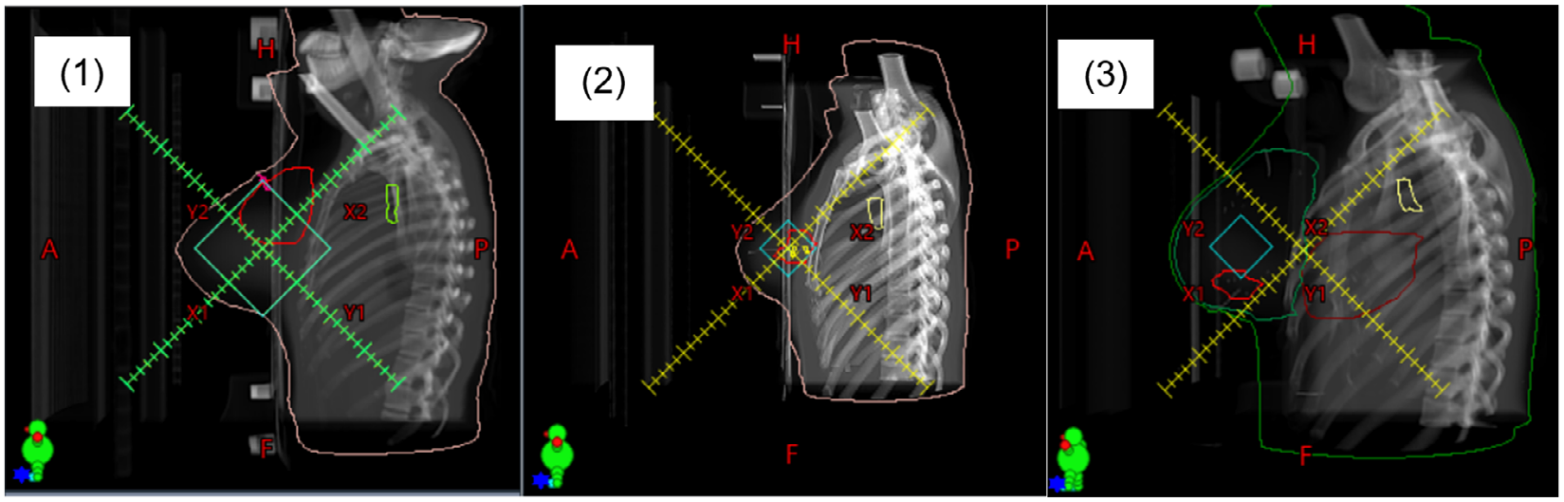
Beam's eye views of three diamond‐shaped light field (DSLF) setups in this study. DSLF‐1 and DSLF‐2 use lateral gantry with the same isocenter as the treatment beam, whereas DSLF‐3 uses the same treatment tangential gantry angle but off‐isocenter to cover the center of the breast for treatment. After the first‐day image‐guided radiation therapy (IGRT), the DSLF is projected on patient, and markers are placed on patient breast skin to reproduce DSLF projection by matching its corners and edges.

Patients were categorized into four groups based on their DSLF setups (Table 1). The DSLF‐1 group included 26 patients using a DSLF of 10 × 10‐cm^2^ FS at direct lateral gantry (90° for right breast and 270° for left) and the same isocenter as treatment tangential beams. The DSLF‐2 group included 20 patients using a DSLF setup similar to that in the first group, except with a FS of 4 × 4 cm^2^. The DSLF‐3 group included 15 patients using a DSLF setup with its light field center along a treatment beam projected on the center of the breast, which was off from the treatment beam isocenter. FSs for the DSLF‐3 group were patient‐specific and varied from 4 × 4 to 6 × 8 cm^2^ with different breast sizes. The fourth group (No DLSF), with 10 patients, relied mainly on skin tattoos and BB markers before kV imaging to reproduce the simulated treatment positions. Beam's eye views of DSLF for the first three groups are shown in Figure 1. All patients, with or without previous DSLF, were set up based on daily kV imaging. The kV‐based couch shift corrections were applied after aligning breast contour, clips, and chest wall on the daily kV image against the DRR at treatment beam's eye view.

**TABLE 1:** Diamond‐shaped light field (DSLF) setups, breast volumes, and couch shift magnitudes

DSLF setups	Patients (*n* = 71)	Breast volume, range (cm^3^)	Daily couch shift avg. ± SD (cm)
(1) FS: 10 × 10 cm^2^, *G* = 90°/270°, @ iso	26	254–3965	0.64 ± 0.4
(2) FS: 4 × 4 cm^2^, *G* = 90° or 270°, @ iso	20	391–2651	0.68 ± 0.3
(3) FS: patient‐specific, *G* = treatment beam angle, off‐iso	15	572–2024	0.80 ± 0.6
(4) No‐DSLF	10	284–2291	1.00 ± 0.6

Abbreviation: avg, average; FS, field size; *G*, gantry angle; SD, standard deviation.

To quantify the benefit of using DSLF, the setup time for the remaining 15 fractions after the first fraction, during which a skin tattoo from the DSLF projection on the breast was created, for 71 patients was exported from ARIA Oncology Information System v15.6 (Varian Medical System, Inc, Palo Alto, CA) via an in‐house script. Setup time, including any skin‐based alignment or IGRT (if any), began from the plan mode‐up until the start of the treatment. In addition, the daily total couch shift correction data, including both vertical and longitudinal shifts following kV‐IGRT of all patient groups, were exported for the analysis of dosimetric effects of different DSLF setups.

The dosimetric effects of using DSLF alignment alone were simulated in a RayStation v8.0a (RaySearch Laboratories, Stockholm, Sweden) treatment planning system by a method described in 2.C. of Xu et al.^13^ By shifting the beam isocenter based on couch shifts between DSLF‐ and IGRT‐based setups, we simulated the dose distribution based on DSLF setup alone. Here, we reasonably assumed that the patient setup based on the final IGRT was representative of the patient position at the initial simulation. The total dosimetric effect of DSLF‐only over the whole treatment course was simulated by accumulating the CT plan dose on day 1, and all the rest 15 shifted virtual fractional doses from day 2 to the last day. This was done to account for the DSLF being generated after the first fraction with the IGRT. Table 2 lists our institutional planning goals for prone breast treatment. Although no specific objectives are included for the chest wall, low chest wall dose is preferred. Simulated doses using DSLF‐only to all of these structures were compared for three different DSLF groups.

**TABLE 2:** Target coverage planning goals and critical organ dose constraints for our prone breast external‐beam radiation therapy

Structure	Planning goals
Whole breast	*D*_95_% ≥ 95% of the prescription dose, *D*_max_ < 110% of the prescription dose
Tumor bed CTV	*D*_95_ ≥ 100% of the prescription dose
Tumor bed PTV	*D*_95_ ≥ 95% of the prescription dose
Heart (if left breast)	*V*_30_ < 5% *D*_mean_ < 3 Gy
Ipsilateral lung	*V*_20_ < 15% (20% also acceptable)

Abbreviation: CTV, clinical target volume; PTV, planning target volume.

## 3 RESULTS

**FIGURE 2:**
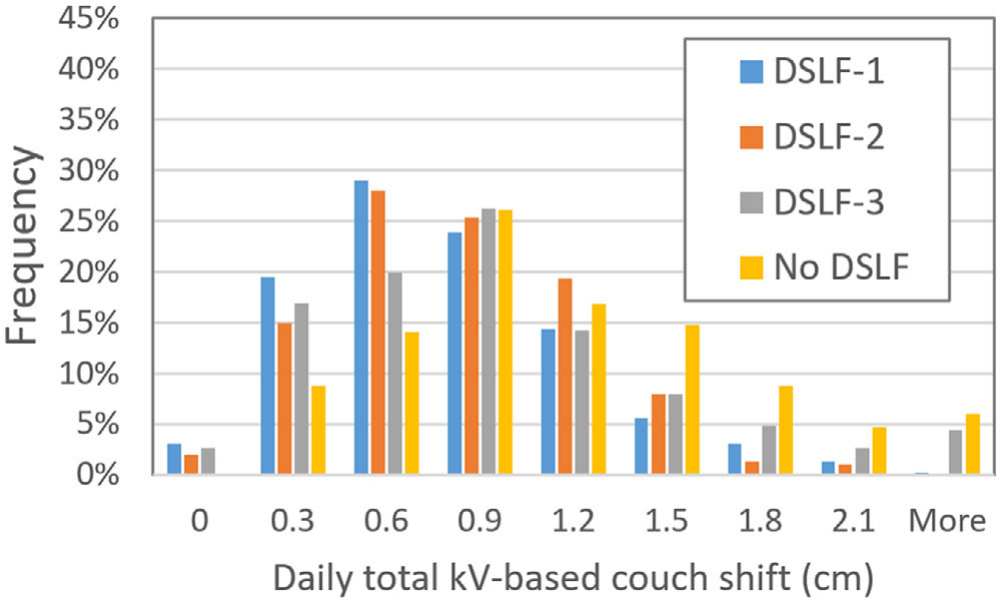
A histogram of daily kV‐based couch shift for four patient groups. The couch shift is square root of the sum of square longitudinal and vertical shifts following 2D kV image‐guided radiation therapy (IGRT) to further match the breast contour, clips, and chest wall with the digitally reconstructed radiograph (DRR) at treatment beam's eye view.

**FIGURE 3:**
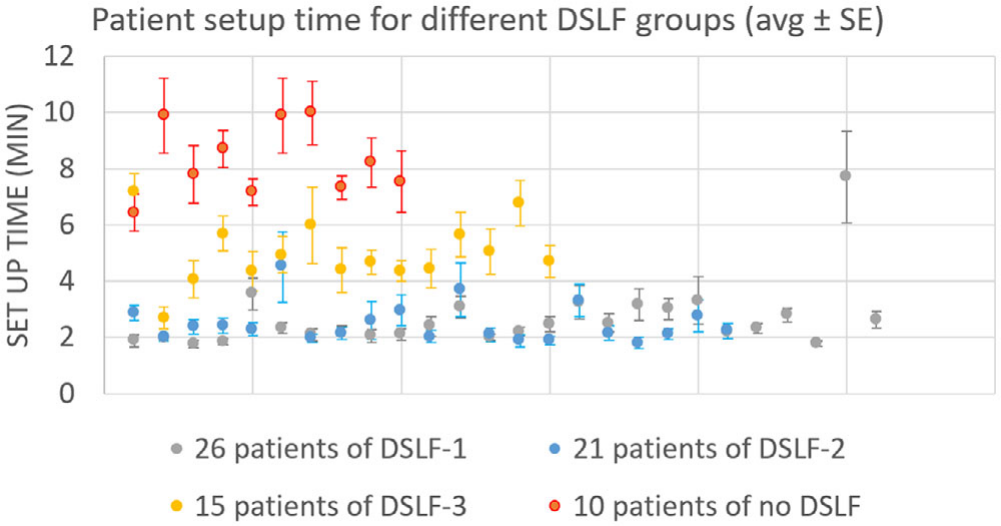
Individual setup time (average ± standard error [SE] of the remaining 15 fractions after the first fraction, during which a skin tattoo from the diamond‐shaped light field [DSLF] projection on the breast was created) for four patient groups. Setup times start from patient plan mode, including patient setup with any skin alignment, image‐guided radiation therapy (IGRT), if any, to the start of the first beam.

As Figure 2 shows, the distributions of daily couch shift magnitudes (the square root sum of squares of daily longitudinal and vertical shifts) are very similar for the DSLF‐1 and DSLF‐2 groups, where the total shift magnitude mainly peaked at the interval of 3–6 mm. For the DSLF‐3 and No‐DSLF groups, the magnitude peaked at the interval of 6–9 mm. The No‐DSLF group was more likely to have a higher magnitude of couch shift corrections, with an average of 10 mm (2–22 mm as 5%–95% percentile). Using DSLF‐reduced couch shift corrections to an average of 7 mm (1–14 mm as 5%–95% percentile) for DSLF‐1 and DSLF‐2 groups, and to an average of 8 mm (1–20 mm as 5%–95% percentile) for DSLF‐3 group. Patient setup times (from patient plan mode‐up to the start of the first treatment beam) in the four groups showed large differences (Figure 3): The average ± standard deviation patient setup times for DSLF‐1 to ‐4 are 2.5 ± 1.8, 2.6 ± 2.1, 5.0 ± 2.9, and 8.2 ± 5.6 min, respectively. For each patient, the setup time can be patient‐specific. The individual average daily setup times ranged from about 2 to 4 min for both DSLF‐1 and ‐2, from 3 to 7 min for DSLF‐3, and from 6 to 10 min for the No‐DSLF group. The standard error of setup times for the No‐DSLF group tended to be larger than those for the DSLF groups.

**FIGURE 4:**
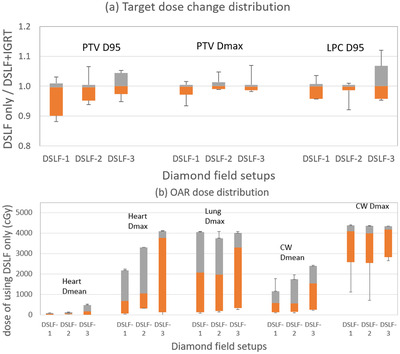
(a) Target dose ratios of diamond‐shaped light field (DSLF)‐only setup to DSLF‐and‐daily‐kV‐image‐guided radiation therapy (IGRT) setup; and (b) OAR doses of using DSLF‐only setup. Upper and lower markers are maximum and minimum values taken over the three DSLF groups; box plot extends from the 10th to 90th percentile; and the lines between gray and orange areas are median values. CW, chest wall

The accumulated dose distributions using three different DSLF setups produced different target coverages and OAR sparings. To compare the target dose of DSLF‐only with the target dose of both DSLF and daily IGRT, their dose ratio distributions for PTV *D*_95_, PTV *D*_max_, and lumpectomy cavity (LPC) *D*_95_ are illustrated in Figure 4a for all the patients. The averaged ratios for these target dose metrics were 0.99, 0.99 to 1.00, and 1.00 to 1.01 for DSLF‐1, DSLF‐2, and DSLF‐3 groups, respectively. A total of 77%, 85%, and 93% of patients from the respective groups maintained PTV *D*_95_ with no more than 3% coverage loss. In general, the DSLF‐3 group, using patient‐specific FS and off‐isocenter setup, yielded a slightly higher target dose, whereas the other DSLF groups with fixed FS and same isocenter setup were more likely to have small target coverage loss relative to DLSF‐3. The absolute doses to heart, lung, and chest wall are shown in Figure 4b. The overall heart *D*_mean_, heart *D*_max_, chest wall *D*_mean_, and lung *D*_max_ of the DSLF‐1 group were lowest and increased from DSLF‐2 to DSLF‐3. The chest wall *D*_max_ was lowest for DSLF‐2 group and increased from DSLF‐1 to DSLF‐3. Compared to the dose delivery with daily IGRT, the failed dose objectives after excluding the IGRT are listed in Table 3. For DSLF‐1 group, 23% failed breast *D*_95_, 4% failed breast *V*_107_, and 10% failed heart mean. For DSLF‐2, 5% failed *D*_max_, 10% failed LPC *D*_95_, and 10% heart *D*_mean_. For DSLF‐3, 13% failed breast *V*_105_ and breast *V*_107_. It shows that DSLF‐1 yields the lowest target coverage but allows for maximum OAR sparing, while DSLF‐3 are more likely to achieve target coverage at the cost of highest OAR dose.

**TABLE 3:** Failed dose objectives of three diamond‐shaped light field (DSLF) groups using DSLF only for prone breast patient setups

DSLF setups	Failed dose objectives
(1) 10 × 10 cm^2^, at iso (26 patients)	6 (23%) fail breast *D*_95_ > 95%, 1 (3.8%) fails breast *V*_107_ < 0, 3 fail (11.5%) heart mean
(2) 4 × 4 cm^2^, at iso (20 patients)	1 (5%) fails breast *D*_max_ < 110%, 2 (10%) fail lumpectomy *D*_95_ > 100% Rx, 2 (10%) fail heart *D*_mean_ < 1 Gy
(3) Off‐iso (15 patients)	2 (13%) fail breast *V*_105_ < 75 cm^3^, 2 (13%) fail breast *V*_107_ < 0

*Note*: Lumpectomy dose metrics failed due to close distance to tangential field border.

**FIGURE 5:**
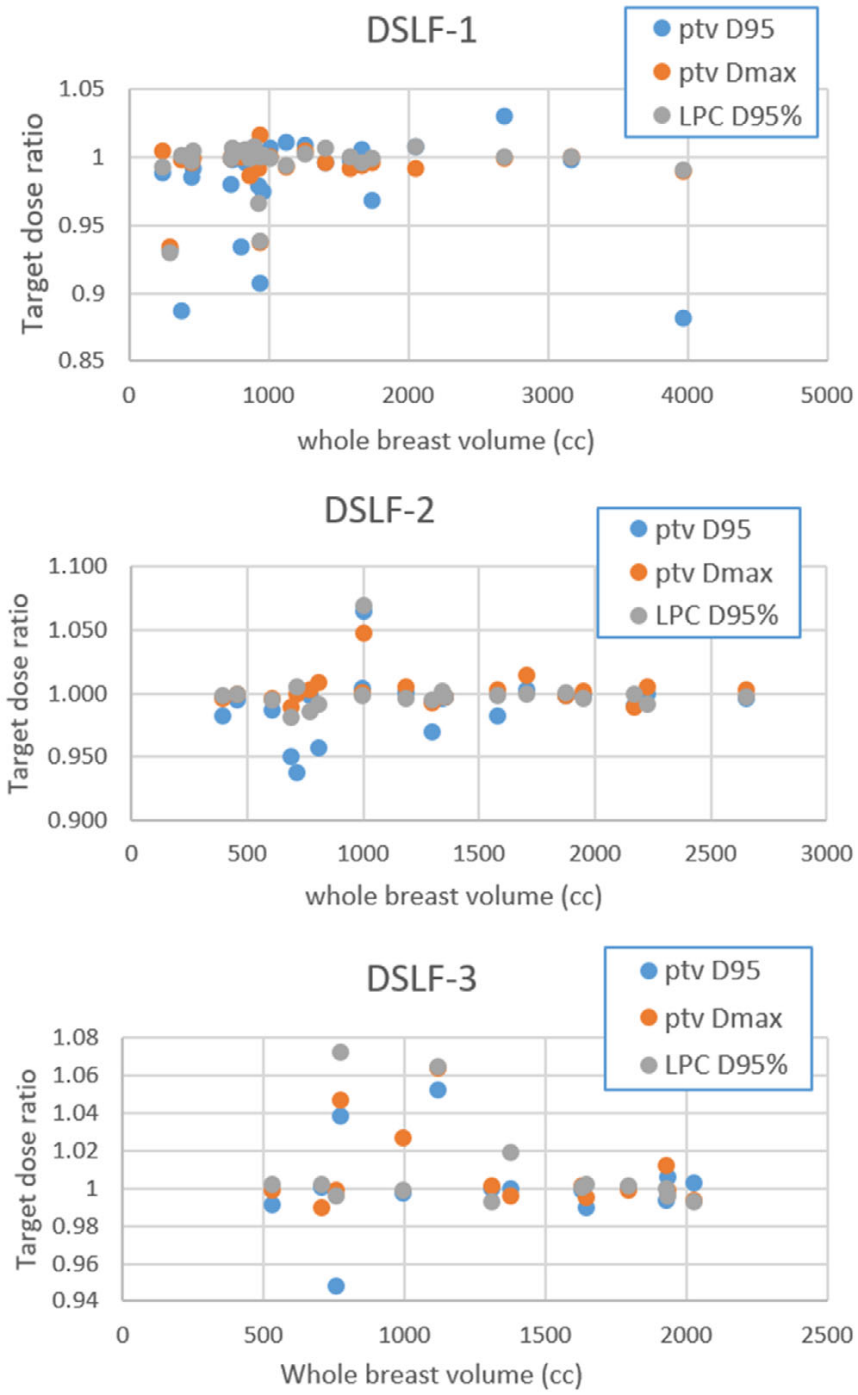
Target dose ratios (diamond‐shaped light field [DSLF]‐only setup vs. DSLF‐and‐daily‐kV‐image‐guided radiation therapy [IGRT] setup) in terms of planning target volume (PTV) *D*_95_, *D*_max_, and lumpectomy *D*_95_ as a function of breast volume for three DSLF setup groups

Figure 5 compares target ratio data (DSLF‐only versus DSLF plus daily kV IGRT) with various breast volumes for three DSLF setups. The target coverage ratio for all target breast *D*_95_, *D*_max_, and lumpectomy *D*_95_ ranged from 0.88 to 1.07, with the majority (>86% data points) within ±3%. Using DSLF only, the OAR dose changes were minimal to lung *V*_20_ and heart *D*_mean_ but could be quite large in the *D*_max_ to lung, heart, and chest wall (−12 to 20 Gy).

## 4 DISCUSSION

This report has evaluated the benefit of DSLF setups for prone breast EBRT by comparing the kV‐based daily couch shifts and patient total setup time among three DSLF groups and one non‐DSLF group. Based on our results, DSLF helps reduce both IGRT correction and patient setup time in comparison with No‐DSLF. In general, DSLF‐1 and ‐2 yield smaller couch shift magnitude and shorter setup time than DSLF‐3. This work also has simulated the dosimetric effects of three different DSLF setups that have been performed across our practices, which should help clinicians select optimal setups for patients per treatment goals. As shown in Figure 1, the off‐center DSLF‐3 projects a light field mainly in the central breast area, whereas DSLF‐1 and DSLF‐2 are closer to the chest wall and other normal tissues. This explains that the DSLF‐3 setup tended to maintain the highest target coverage at the cost of the highest OAR dose, whereas DSLF‐1 and DSLF‐2 (using the same isocenter as the treatment beams) resulted in more OAR sparing for heart, lung, and chest wall. More specifically, DSLF‐1 was most efficient in sparing heart and chest wall, DSLF‐2 had the lowest lung *D*_max_, On the other hand, the wide range of target and OAR dose in each DSLF group indicates that DSLF dosimetric effect can be quite patient‐specific. Our findings provide useful information to optimize prone breast treatment setup and/or standardize the DSLF setup across clinical sites to achieve a specific treatment goal.

The dosimetric effect of DSLF FS changes with the breast volume was investigated. Figure 5 shows the target dose ratios for DSLF‐1 of 10 × 10 cm^2^ FS as a function of breast volume of 245–3965 cm^3^, in regards to DSLF‐2 and DSLF‐3 groups of smaller FS. For the two patients of the DSLF‐1 group that had PTV *D*_95_ lower than 90%, one breast volume was <500 cm^3^, whereas the other was about 4000 cm^3^. It shows a weak correlation (correlation coefficient <0.4) between DSLF FS and breast target coverage. Therefore, breast volume is not a key factor that determines the DSLF FS for the most efficient prone breast setup to cover the target.

One of the limitations of this work is the lack of 3D IGRT to consider organ deformation. Ideally, CBCT data from daily treatment should be used to track and optimally deliver the planned doses to target and OAR structures, Piechowska^18^ reported on a study in which patients were set up based on the standard constant source to surface distance verification and lateral light fields (same as DSLF) with weekly port films and daily post‐setup CBCT. In that work, the initial setup for 2 out of 11 patients failed to deliver the dose to at least 90% of the tumor bed, but all other patients had accurate initial setups. Even for the bad cases, CBCT images still help improve treatment localization.

Another limitation of this study is the degrees of freedom (DOF) of couch corrections. DSLF helps reduce couch correction magnitude and may waive the need for rotational corrections. However, couch corrections on a higher DOF may further improve interfractional reproducibility for the prone breast treatment setup. Although the most advanced 6‐DOF couch provides both translational and rotational (roll, pitch, and yaw) setup corrections, its clinical benefit relative to the conventional 3‐ or 4‐DOF is debatable for non‐stereotactic‐radiosurgery procedures.^19^ It will be interesting to address this topic in the future.

## 5 CONCLUSION

This work evaluates the benefit of using DSLF for prone breast patient setup. In general, DSLF improves setup efficiency by significantly reducing patient setup time. DSLF also yields smaller IGRT corrections. If IGRT is limited, different DSLF setups provide different tradeoffs in target coverage and OAR dose sparing. Our findings will help design efficient DSLF setups and optimize the prone breast setup workflow.

## AUTHOR CONTRIBUTION

We confirm that all coauthors contributed this work and agreed with the submission of this manuscript to JACMP.
